# Implementing an electromagnetic tracking navigation system improves the precision of endoscopic transgastric necrosectomy in an ex vivo model

**DOI:** 10.1038/s41598-024-60647-w

**Published:** 2024-05-02

**Authors:** Anna Fichtl, Alaan Sheikhani, Martin Wagner, Alexander Kleger, Martin Müller, Niklas Sturm, Benjamin Walter, Alfred Michael Franz

**Affiliations:** 1 Department of Internal Medicine I, University Hospital Ulm, Albert-Einstein-Allee 23, 89081 Ulm, Germany; 2 Endoscopic Research Unit, University Hospital Ulm, Albert-Einstein-Allee 23, 89081 Ulm, Germany; 3 Institute for Medical Engineering and Mechatronics, University of Applied Sciences Ulm, Albert-Einstein-Allee 53-55, 89081 Ulm, Germany; 4 Institute of Molecular Oncology and Stem Cell Biology, University Hospital Ulm, Albert-Einstein-Allee 23, 89081 Ulm, Germany; 5 Division of Interdisciplinary Pancreatology, Department of Internal Medicine I, University Hospital Ulm, Albert-Einstein-Allee 23, 89081 Ulm, Germany; 6 Division of Intelligent Medical Systems, German Cancer Research Center (DKFZ), Im Neuenheimer Feld 223, 69120 Heidelberg, Germany

**Keywords:** Pancreatitis, Biomedical engineering

## Abstract

Endoscopic transgastric necrosectomy is crucial in the management of complications resulting from necrotizing pancreatitis. However, both real-time and visual-spatial information is lacking during the procedure, thereby jeopardizing a precise positioning of the endoscope. We conducted a proof-of-concept study with the aim of overcoming these technical difficulties. For this purpose, a three-dimensional (3D) phantom of a stomach and pancreatic necroses was 3D-printed based on spatial information from individual patient CT scans and subsequently integrated into a silicone torso. An electromagnetic (EM) sensor was adjusted inside the endoscope´s working channel. A software interface enabled real time visualization. The accuracy of this novel assistant system was tested ex vivo by four experienced interventional endoscopists who were supposed to reach seven targets inside the phantom in six different experimental runs of simulated endoscopic transgastric necrosectomy. Supported by endoscopic camera view combined with real-time 3D visualization, all endoscopists reached the targets with a targeting error ranging between 2.6 and 6.5 mm in a maximum of eight minutes. In summary, the EM tracking system might increase efficacy and safety of endoscopic transgastric necrosectomy at the experimental level by enhancing visualization. Yet, a broader feasibility study and further technical improvements are mandatory before aiming at implementation into clinical setting.

## Introduction

Necrotizing pancreatitis is a potentially life-threatening condition emerging in up to 20% of patients affected by acute pancreatitis. The preferred treatment strategy for patients with large or infected pancreatic necroses is a step-up approach including minimally invasive endoscopic interventions such as the endoscopic transmural necrosectomy^1,2^. Endoscopic necrosectomy can be performed transgastrically, transduodenally or via any other access route entering the necrosis bearing retroperitoneum, but its usage should be limited to referral centres because of high risks of bleeding (18%), pancreatic fistula development (5%), and perforation (4%)^3,4^. While major risk complications such as bleeding can be decreased by employing endoscopic ultrasound (EUS)‐guidance to access the necrotic tissue^5^, subsequent necrosectomy frequently necessary within the opened necrotic cavity lacks the accurate visual and most importantly 3D position of the endoscope. However, such information is crucial to prevent complications such as large vessel injury or accidental entry of the abdominal cavity. Therefore, we conducted a proof-of-concept study striving for the development of a 3D-assistance system/approach to reduce procedure time and enhance accuracy of endoscopic necrosectomy.

## Procedures

### Study design and participants

We created an ex vivo model consisting of 3D-printed replicas of stomach, pancreas, and necroses. Additionally, we equipped an endoscope with an electromagnetic (EM) sensor that can be localized in relation to an EM tracking system respectively EM field generator. This setup was used for experimental runs of endoscopic transgastric necrosectomy enhanced by an EM navigation system. Four experienced interventional endoscopists in our university medical centre performed the procedure.

### Investigational setting

1. 3D phantom of pancreatic necroses

**Figure 1:**
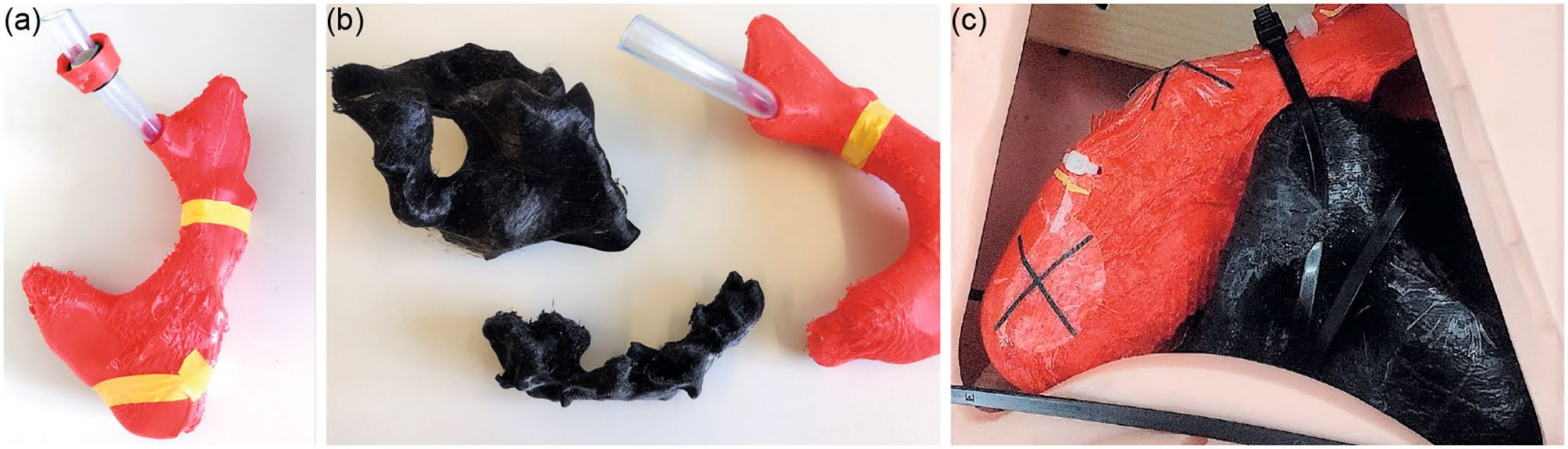
(**a**) 3D-printed model of a stomach based on patient data. The stomach model was previously cut longitudinally, marked with 5 CT markers on the inside, and then reassembled with yellow tape. Additionally, a transparent tube simulating the esophagus is connected to it. (**b**) 3D-printed pancreatic necroses (black) based on patient data. For size comparison, a section of the stomach model is located to the right of it. (**c**) 3D-printed models of the stomach (red) and pancreatic necroses (black) integrated into a silicone torso. To avoid displacement of the necroses during experimental necrosectomy, the necroses were fixed to the stomach model with cable ties. The two transparent stickers with black crosses attached to the outside of the stomach model are the registration markers.

**Figure 2:**
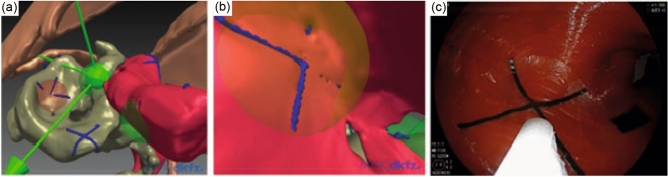
(**a**) 3D overall navigation mode during experimental transgastric necrosectomy, generated by a custom software based on the Medical Imaging Interaction Toolkit (MITK) Plugin and provided by the German Cancer Research Center. The blue crosses correspond to the targets in form of CT-markers. Two extramural targets are visible inside the necrotic cavity. In this figure, only two out of the total of five CT markers present in the stomach are visible. Red: stomach. Brown: inner wall of the silicone torso. Gray-green: pancreatic necroses. Blue crosses: targets. Green sphere and arrows: position and orientation of the tip of the endoscope. (**b**) Virtual camera view from the tip of the endoscope inside the stomach. (**c**) Corresponding real camera view of the endoscope. Black: CT marker in real camera view.

Computer tomography (CT) scans of one patient suffering from necrotizing pancreatitis were manually processed into 3D segments of the stomach, the necrotic areas, and surrounding organs in the Medical Imaging Interaction Toolkit (MITK), an open-source software for volume and surface visualization^6^. The 3D segmented models were edited via the computer-aided design (CAD) and computer-aided manufacturing (CAM) software Fusion360 (Autodesk Inc., San Rafael, California, USA) in combination with MeshLab (v2021.07, ISTI-CNR, Pisa, Italy) and printed by means of a Fused filament fabrication (FFF) Creality 3D printer (Ender-5 S1, Creality, Shenzhen, China). The 3D printing process was controlled through the CURA software. After 3D printing, the different parts of the phantom were placed into a torso manufactured from silicone (Fig. 1). Seven target CT markers (ReBeck CT-Marker "cross black", FOBECK GbR, Tiefenbach, Germany) were placed inside the phantom as shown in Fig. 2: Five points were located on the inside of the stomach wall to simulate potential access routes to the necrotic cavity whereas the other two on the necrotic cavity (extramural). Six additional markers were used for registration, from which three were placed on the outer wall of the stomach, two on the necrosis and one inside the phantom's torso. CT markers are originally used for precise delineation of a target volume in CT examinations, known for causing minimal artifacts in imaging, being easy to apply, and remaining immovable once attached to the surface^7^.

2. Electromagnetic tracking system and registration

EM tracking was conducted with the Aurora tracking system (Northern Digital Inc. (NDI), Waterloo, Canada). This system consists of an EM sensor featuring six degrees of freedom and a diameter of 1.3 mm, a tabletop field generator, and a processing control unit. The EM sensor was inserted in the working channel of a gastroscope (GIF-H190; Olympus Medical Systems, Tokyo, Japan; EG-600 Fujifilm Endoscopy, Tokyo, Japan) and the tip of the sensor was fixed when overtopping the tip of the endoscope by 5 mm so that the sensor tip was visible through the camera of the endoscope as shown in Fig. 2c). The navigation during endoscopy occurred via a conventional video monitor view which was extended by a virtual 3D visualization of the endoscope and its surrounding structures inside the phantom as shown in Fig. 2a,b. The virtual scene was built with the help of the EM tracking system and a custom software based on the MITK using the image guided therapy (IGT) modules. To enable 3D visualization, a CT was acquired before the experiment and the anatomical structures were segmented manually to generate 3D models of the scene. The virtual scene was registered to the tracking space by a point-based transform according to Horn et al.^8^. Therefore, three marker positions within tracking space were approached externally with an EM pointer. The centres of the corresponding markers were indicated in the CT image space.

### Experimental transgastric necrosectomy enhanced by electromagnetic tracking

The phantom was placed on a wooden plate located 11 cm above the NDI tabletop field generator and fixed to prevent unexpected movements. Subsequently, the phantom served as a model for endoscopic transgastric necrosectomy. Four endoscopists with several years of experience in performing ERCP and endoscopic necrosectomy were involved in the study. They joined efforts to reach the seven targets with the tip of the endoscope during six experimental runs of transgastric necrosectomy. During the examination two different modes were available: an overall navigation mode representing a virtual external view of the endoscope and its surroundings (Fig. 2a) and a sensor view navigation mode simulating the endoscopic camera view (Fig. 2b). The virtual scene created in the overall navigation mode could be adjusted freely to different view angles as required.

In the first run, only the camera view of the endoscope was used, while during the second run, only the virtual assistant system was available. For all subsequent runs, a combination of endoscopic as well as the virtual view were used. In all cases, the camera view (Fig. 2c) was used to place the sensor tip exactly in the centre of the real marker. The distance between sensor and marker position (from registered CT space) in the virtual scene was subsequently used as a measure of system accuracy. After completing the experimental transgastric necrosectomy, the four investigators were interrogated to evaluate the assistance system in terms of usefulness, realistic implementation, and accuracy level during endoscopic intervention. A grading for each assessed parameter from one to five, in which ‘five’ was delineated as the best and ‘one’ corresponds to the worst category for every parameter.

### Outcome definitions

The overall navigation error as primary outcome was defined as the Euclidian distance between the seven target positions in the virtual scene and the corresponding positions in the physical phantom as described before. The secondary outcomes included the procedure time and the fiducial registration error (FRE). The FRE is used in medical imaging to assess the accuracy of aligning or registering different image datasets and it is defined as the root-mean-square error in fiducial alignment between virtual and real space^9^. Furthermore, the investigators´ feedback concerning usefulness, realistic implementation, and accuracy level in endoscopic intervention was evaluated.

## Results

All endoscopists managed to reach the seven targets within the phantom with a mean navigation error ranging between 2.6 and 6.5 mm and a procedure time of 3:31 and 7:15 min:s. For investigator 1, both the mean navigation error and procedure time were lowest when the experimental investigation was performed with a combination of endoscopic camera view and the virtual assistant system. In the comparison of run 1 (endoscopic camera view only) with run 3–6 (combination of endoscopic camera view and the virtual assistant system), the mean navigation error was lower when using endoscopic camera view, while the procedure time was lower when the combination of endoscopic camera view and the virtual assistant system was used (Table 1).

**Table 1:** Navigation error, fiducial registration error and procedure time of six experimental runs of endoscopic transgastric necrosectomy relying on conventional endoscopic camera view and a virtual assistance system.

Experimental run		1	2	3	4	5	6
Investigator		1	1	1	2	3	4
Used system		EC	AS	EC, AS	EC, AS	EC, AS	EC, AS
Navigation error (mm)	Target 1	6.5	4.9	2.8	0.6	3.2	3.0
	Target 2	4.7	6.3	0.7	6.2	5.1	5.8
	Target 3	5.4	9.7	1.4	3.6	6.4	6.4
	Target 4	0.9	1.6	3.1	2.5	0.9	3.2
	Target 5	5.3	8.9	2.9	2.7	2.1	6.7
	Target 6	2.5	5.9	3.0	1.8	1.7	6.2
	Target 7	4.1	6.7	5.5	1.0	1.0	7.0
	Mean	4.2	6.3	2.8	2.6	2.9	6.5
FRE (mm)§		1.2	1.2	1.2	1.1	1.1	1.2
Time (min:s)		3:31	4:56	3:12	7:15	5:19	5:10

*EC* endoscopic camera, *AS* assistance system, *FRE* fiducial registration error.

**Figure 3:**
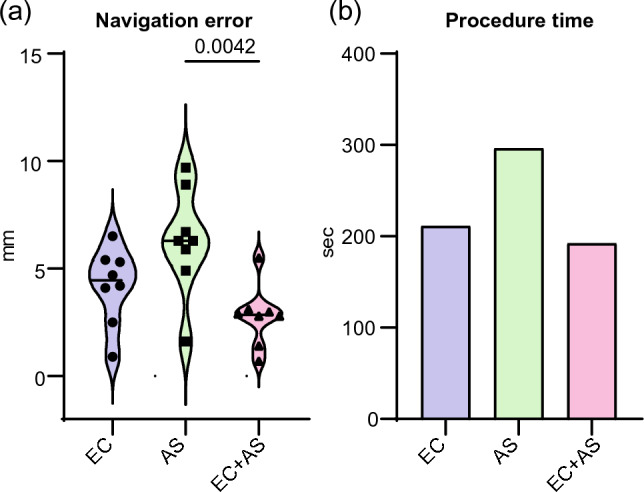
(**a**) Performance of investigator 1 during experimental endoscopic necrosectomy: Navigation errors in mm (y-axis) depending on the experimental condition for reaching the seven targets (x-axis). Each data point corresponds to one of the seven targets. The p-value for the navigation error when using EC + AS compared to AS alone is reported as 0.0042 and was calculated using a one-way analysis of variance. (**b**) Procedure time in seconds (y-axis) of the three experimental sessions of endoscopic necrosectomy performed by investigator 1 depending on the experimental condition for reaching the seven targets (x-axis). *EC* endoscopic camera, *AS* assistance system, circle, square, and triangle in black = circle = navigation errors while reaching the seven targets during the first (circle), the second (square) and the third (triangle) experimental run. Circle, square, and triangle in red = mean navigation error after first (circle), the second (square) and third (triangle) experimental run.

For all experimental runs, the FRE was in the range of 1.1 and 1.2 mm. Investigator 1 performed three experimental runs of endoscopic necrosectomy using either the endoscopic camera view or the assistance system or a combination of both types of visualization. In this setting, the best results were obtained using the combination of endoscopic camera view and assistance system (Fig. 3).

The investigators rated the categories usefulness, realistic implementation, and accuracy level in endoscopic intervention with 4.75/5, 3.75/5, and 4.5/5 respectively (Table 2).

**Table 2:** Subjective evaluation of the test runs, employing an endoscopic camera view in conjunction with a 3D navigation model, regarding the categories of usefulness, realistic implementation in clinical practice, and accuracy level in endoscopic intervention. The assessments were carried out by the four investigators, with a rating scale from 1 indicating the poorest to 5 points denoting the highest possible rating.

Investigator	Assessment criterion “Usefulness”	Assessment criterion “Realistic implementation”	Assessment criterion “Accuracy level in endoscopic intervention”
1	5/5	3/5	4/5
2	5/5	4/5	5/5
3	4/5	3/5	4/5
4	5/5	5/5	5/5
Mean	4.75/5	3.75/5	4.5/5

## Discussion

We demonstrated here the feasibility of combining endoscopic necrosectomy with a virtual assistance system. The EM tracking system used in our study allowed an accurate localization of the endoscope and its surrounding. Yet, the results are limited given some technical challenges: First, the placement of the EM sensor inside the working channel of the endoscope precludes simultaneous tracking and interventional actions such as necrosectomy. Thus, endoscopes require further adjustments for clinical application, as previously demonstrated for flexible endoscopes in other applications such as the case of the ScopeGuide system (Olympus K.K., Tokyo, Japan) for colonoscopy^10^. Second, the features of the inner surface of the 3D printed phantom did not yet reach the true-to-life accuracy due to a rather rough surface. The use of other printing technologies such as Stereolithography (SLA) printing instead of FFF printing as employed in the current study, may lead to a better surface quality^11^. To make the 3D model of the necrotic cavity even more realistic, in the future, surrounding blood vessels or vessels passing through the necrotic cavity could be added. Third, the setup presented in this study aims at minimizing navigation errors which must be considered according to the clinical assessment of the EM tracking system: In a clinical setting, registration errors occur due to movements of the patient or slipping of registration markers. Dynamic errors result from the constant movement of the sensor. Furthermore, interaction with different metal objects such as stents, might cause undesired magnetic field distortion^12^. In endoscopic necrosectomy, stents containing Nitinol are commonly used. Nitinol is a non-ferromagnetic material^13^, so no significant disruptions of the electromagnetic field are expected from Nitinol stents. Currently, however, there is no available data on the extent to which Nitinol stents can impact the electromagnetic field.

For this initial study, we employed a rather straight-forward approach of point-based registration, yet which requires a relatively large amount of interaction. However, the implementation in clinics routine mandates a robust fully automatic registration as e.g. presented by Mittmann et al.^14^. Given the availability in the MITK, we envision a rapid integration into the future prototype. Similarly, segmentation of anatomical structures in CT was conducted manually here can be upgraded in future upon involving Deep Learning methods^15^. Their initial insufficient robustness for fully automated use could be substantiated by direct volume visualization.

Concerning the mean values of navigation errors throughout demonstrations, investigator 1 showed the highest error with 6.3 mm during run 1 (with assistant system and without the endoscopic camera), while scoring in the mid-range of accuracy upon employing the endoscope camera per se. At the same time, a combination of endoscope camera with assistant system scored the best with 2.8 mm (Fig. 3a). This suggests that relying alone on the assistant system is rather inferior/disadvantageous in comparison to the conventional visualization with just an endoscope camera. However, the fact that investigator 1 performed best during the experimental run 3 (with assistant system and endoscopic camera) might also reflect the skills acquired during training. Notably, investigators 2 and 3 produced similar navigation errors as investigator 1 without a preceding training. In contrast, investigator 4 produced significantly worse navigation errors than the first 3 investigators. This was mainly ascribed to the fact that investigator 4 struggled most with the surface quality of the phantom. Our investigations indicate that repeated training may be crucial in flattening the level of accuracy between investigators. In line with this, a recent clinical study on the use of a 3D navigation system for liver surgery reported similar findings. The study involved 26 experienced liver surgeons who considered 3D-printed models beneficial for surgical procedures after using a novel mixed reality navigation system^16^. Furthermore, the majority of study participants (85%) indicated that 3D models are useful for determining safer surgical paths and for training inexperienced surgeons.

As for the time required by each investigator to complete the experimental run, we documented that the same investigator (experimental run: 1, 2, 3) using the combination of two systems achieved the shortest procedure time (Fig. 3b). The fact that run 2 took longer than run 1 can be explained by the need for some adaptation to the use of the virtual navigation system. Compared to investigators 2, 3 and 4, the familiarity of investigator 1 with the system might be an important determinant for the experimental outcome.

Notably, the virtual assistance system developed in this study served as an add-on and not as a substitute for the conventional endoscopic camera view during experimental endoscopic necrosectomy.

An important technological innovation aimed at enhancing the safety of the endoscopic necrosectomy procedure is the so-called EndoRotor System. It enables the debridement of pancreatic necroses under direct endoscopic visualization^17^. Although few intraprocedural adverse events have been described with this new automated resection system so far^18^, cases of perforation during examinations with EndoRotor are known^17,19^. This underscores the need for further improvements, such as our developed 3D navigation system, to further minimize severe complications like perforation and bleeding in the future.

In summary, we showed for the first time that combining transgastric necrosectomy with a virtual assistant system based on EM tracking is technically feasible. Its utilization could help conserve X-ray radiation in the future and further enhance the safety of necrosectomy. The novel assistant system developed in this study needs to undergo further evaluation in larger studies, particularly regarding its accuracy, procedure time, and potential as a training tool for inexperienced investigators. Furthermore, technical improvements of the EM tracking systems and a comparison of EM tracking with optical tracking and other methods such as X-ray fluoroscopy are necessary before clinical implementation.

### Supplementary Information

Supplementary Information.

Supplementary Video 1.

Supplementary Video 2.

## Data Availability

All relevant data collected within the scope of the study have been listed in this article. Upon justified request, A. F. can provide data on preliminary experiments regarding the flexibility and measurement accuracy of the electromagnetic probe before its integration into the working channel of the endoscope.

## References

[1] Trikudanathan G (2019). Current concepts in severe acute and necrotizing pancreatitis: An evidence-based approach. Gastroenterology.

[2] Van Santvoort HC (2010). A step-up approach or open necrosectomy for necrotizing pancreatitis abstract. N. Engl. J. Med..

[3] Baron TH, DiMaio CJ, Wang AY, Morgan KA (2020). American gastroenterological association clinical practice update: Management of pancreatic necrosis. Gastroenterology.

[4] Arvanitakis M (2018). Endoscopic management of acute necrotizing pancreatitis: European Society of Gastrointestinal Endoscopy (ESGE) evidence-based multidisciplinary guidelines. Endoscopy..

[5] Jha AK, Goenka MK, Kumar R, Suchismita A (2019). Endotherapy for pancreatic necrosis: An update. JGH Open..

[6] Nolden M (2013). The medical imaging interaction toolkit: Challenges and advances: 10 years of open-source development. Int. J. Comput. Assist. Radiol. Surg..

[7] Habermehl D (2013). Evaluation of different fiducial markers for image-guided radiotherapy and particle therapy. J. Radiat. Res..

[8] Horn BKP (1987). Closed-form solution of absolute orientation using unit quaternions. J. Opt. Soc. Am..

[9] Fitzpatrick, J. M. Fiducial registration error and target registration error are uncorrelated. In *Medical Imaging 2009: Visualization, Image-Guided Procedures, and Modeling* vol. 7261 726102 (SPIE, 2009).

[10] Mark-Christensen A, Brandsborg S, Iversen LH (2014). Magnetic endoscopic imaging as an adjuvant to colonoscopy: A systematic review and analysis of randomized controlled trials. Endoscopy..

[11] Özgür Ö (2021). Comparison of the surface quality of the products manufactured by the plastic injection molding and SLA and FDM method. Uluslararası Muhendislik Arastirma ve Gelistirme Dergisi.

[12] Franz AM (2014). Electromagnetic tracking in medicine—A review of technology, validation, and applications. IEEE Trans. Med. Imaging.

[13] Stoeckel, D., Pelton, A. & Duerig, T. Self-expanding Nitinol stents for the treatment of vascular disease. In *Shape Memory Alloys for Biomedical Applications* 237–256 (Elsevier, 2009). 10.1533/9781845695248.2.237.

[14] Mittmann BJ (2022). Reattachable fiducial skin marker for automatic multimodality registration. Int. J. Comput. Assist. Radiol. Surg..

[15] Isensee F, Jaeger PF, Kohl SAA, Petersen J, Maier-Hein KH (2021). nnU-Net: A self-configuring method for deep learning-based biomedical image segmentation. Nat. Methods.

[16] Shahbaz M (2023). Mixed reality navigation training system for liver surgery based on a high-definition human cross-sectional anatomy data set. Cancer Med..

[17] Olsen GA, Schmidt PN, Novovic S, Hansen EF, Karstensen JG (2023). Novel powered 5.0 mm endoscopic debridement catheter for endoscopic transmural necrosectomy of pancreatic walled-off necrosis: A case series of consecutive patients from a tertiary referral center (with video). Gastrointest. Endosc..

[18] Binda C (2023). Direct endoscopic necrosectomy of a recurrent walled-off pancreatic necrosis at high risk for severe bleeding: A hybrid technique using a dedicated device. Diagnostics.

[19] Gotink AW, Peters Y, Bruno MJ, Siersema PD, Koch AD (2022). Nonthermal resection device for ablation of Barrett’s esophagus: A feasibility and safety study. Endoscopy.

