# Supplementary material for: Implementing an electromagnetic tracking navigation system improves the precision of endoscopic transgastric necrosectomy in an ex vivo model
Source: Sci Rep. 2024 May 2;14:10055. doi: 10.1038/s41598-024-60647-w (PMC11066121; doi:10.1038/s41598-024-60647-w)
Supplement: Supplementary file 1 — Supplementary Information. [file 41598_2024_60647_MOESM1_ESM.docx]

**Implementing an electromagnetic tracking navigation system improves the precision of endoscopic transgastric necrosectomy in an ex vivo model.**

Anna Fichtl^1,2,*^, Alaan Sheikhani^3^, Martin Wagner ^1^, Alexander Kleger^4,5^, Martin Müller^1^, Niklas Sturm^1,2^, Benjamin Walter^1,2,^ †, Alfred Michael Franz^3,6,^ †

^1^ Department of Internal Medicine I, University Hospital Ulm, Albert-Einstein-Allee 23, 89081 Ulm, Germany

^2^ Endoscopic Research Unit, University Hospital Ulm, Albert-Einstein-Allee 23, 89081 Ulm, Germany

^3^ Institute for Medical Engineering and Mechatronics, University of Applied Sciences Ulm, Albert-Einstein-Allee 53-55, 89081 Ulm, Germany

^4^Institute of Molecular Oncology and Stem Cell Biology, University Hospital Ulm, Albert-Einstein-Allee 23, 89081 Ulm, Germany

^5^ Division of Interdisciplinary Pancreatology, Department of Internal Medicine I, University Hospital Ulm, Albert-Einstein-Allee 23, 89081 Ulm, Germany

^6^ Division of Intelligent Medical Systems, German Cancer Research Center (DKFZ), Im Neuenheimer Feld 223, 69120 Heidelberg, Germany

† contributed equally

**Supporting information**

**Video 1:** Real-time application of the electromagnetic assistance system in endoscopic camera view mode. The tip of the endoscope is located in the 3D plastic model of the stomach and is moved towards one of the five targets present in the stomach. On the right: Real endoscopic camera view. To ensure that the investigators could accurately reach the targets with the sensor tip, the sensor tip extended 5 millimeters beyond the endoscope tip. On the left: Virtual endoscopic camera view of the same scene.

**Video 2:** Real-time application of the electromagnetic assistance system in general navigation mode. The tip of the endoscope was positioned at the transition from the stomach to the necrotic cavity and moved into the necrotic cavity. On the right: Real endoscopic camera view of the stomach (skin-colored) and the necrotic cavity (black). On the left: General navigation mode. This virtual mode provides an external view of the 3D models of the stomach (red), necrotic cavity (gray-green), the placed targets (crosses), and the endoscope tip, as well as their spatial relationships to each other. The three-dimensional orientation of the endoscope tip is illustrated by arrows.
